# Experimental error analysis of biomechanical phenotyping for stalk lodging resistance in maize

**DOI:** 10.1038/s41598-023-38767-6

**Published:** 2023-07-27

**Authors:** Joseph DeKold, Daniel Robertson

**Affiliations:** Department of Mechanical Engineering, University of Idaho, 875 Perimeter Drive, MS 0902, Moscow, ID 83844-0902 USA

**Keywords:** Mechanical engineering, Plant sciences, Plant breeding

## Abstract

Stalk lodging destroys between 5 and 25% of grain crops annually. Developing crop varieties with improved lodging resistance will reduce the yield gap. Field-phenotyping equipment is critical to develop lodging resistant crop varieties, but current equipment is hindered by measurement error. Relatively little research has been done to identify and rectify sources of measurement error in biomechanical phenotyping platforms. This study specifically investigated sources of error in bending stiffness and bending strength measurements of maize stalks acquired using an in-field phenotyping platform known as the DARLING. Three specific sources of error in bending stiffness and bending strength measurements were evaluated: horizontal device placement, vertical device placement and incorrect recordings of load cell height. Incorrect load cell heights introduced errors as large as 130% in bending stiffness and 50% in bending strength. Results indicated that errors on the order of 15–25% in bending stiffness and 1–10% in bending strength are common in field-based measurements. Improving the design of phenotyping devices and associated operating procedures can mitigate this error. Reducing measurement error in field-phenotyping equipment is crucial for advancing the development of improved, lodging-resistant crop varieties. Findings have important implications for reducing the yield gap.

## Introduction

It is estimated that grain crops account for over 50% of the average person’s caloric consumption^1,2^. The global demand for grain continues to grow each year as the global population increases. For example, the global corn export market grew by 7.2% annually between 2012 and 2021^3^. In the United States, maize (*Zea mays*) exports totaled $9.2 billion in 2021, which was a 20% increase ($1.6 billion) from 2020^4^. Meeting the global demand for grain is becoming increasingly difficult due to numerous factors including: climate variability, urbanization, increasingly frequent extreme weather events and drought^5,6^. Reducing the yield gap and improving agronomic efficiencies will be necessary to continue meeting the global food, fuel and fiber demand of the future^7^.

The problem of stalk lodging (breakage of the plant stem prior to harvest) significantly reduces the annual yield of vital grain crops like maize, rice (*Oryza sativa)* and wheat (*Triticum)*. For example, lodging is estimated to reduce cereal crop yields by up to 20%, resulting in billions of dollars of lost grain annually^3,8–10^. Reducing the overall yield losses in maize by just 1% would provide an additional 6.9 million metric tons of corn commodity in the United States alone^3^. Lodging is a highly plastic and complex phenotype that is ultimately dependent upon numerous external and internal factors that can vary both spatially and temporally. External factors include crop management practices, weather, disease, and insect pressure^11–13^. Internal factors include numerous mechanical properties of the plant (e.g., bending strength, stalk geometry) that have genetic underpinnings^14–19^. The increasingly variable and extreme global climate is expected to further aggravate lodging incidence and related economic losses in the future^20^. The complex multi factor nature of stalk lodging makes it challenging to evaluate and assess in genetic and breeding studies.

Selective breeding has reduced lodging rates and increased yield/hectare^21–23^. However, despite such advances, stalk lodging is still a major unsolved agronomic problem. For over a century, groups have been developing devices and techniques to quantify the lodging resistance of crops to aid in selective breeding efforts (e.g., Refs.^14,16,17,24–37^). These devices have been used in numerous studies to investigate the biomechanical response of plant stems^15,18,38–47^. However, best practices and clear methodological details for many of these devices are lacking in scientific literature. The purpose of this study is to conduct an experimental error analysis of a commonly used electromechanical field-deploying device which acquires measurements of stalk bending strength and stalk bending stiffness known as the DARLING^27,48^.

**Figure 1:**
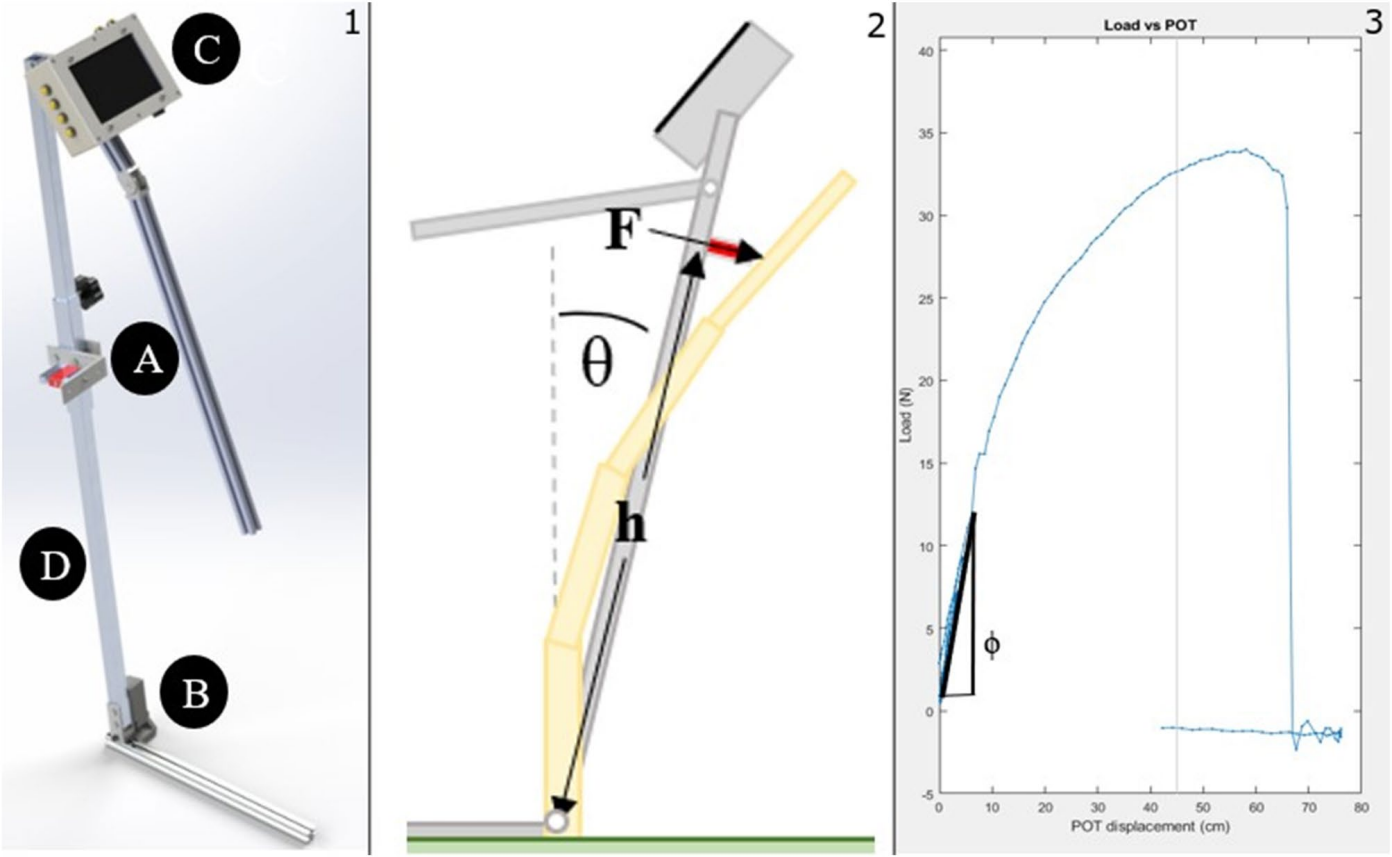
(Panel 1) Like many field-based devices, the DARLING uses force sensors (**A**) and angular rotation sensors (**B**) to measure applied force and angular displacement. A user interface (**C**) enables editing of recorded data and appending of metadata to each test sample. The sensors and user interface are mounted to a supportive skeleton (**D**). (Panel 2) Schematic illustrating load cell height, applied force and angular displacement. (Panel 3) A typical force vs displacement curve generated while deforming a maize stalk. Bending stiffness is calculate based on the slope of the initial, linear portion of the data curve (Φ), and bending strength is calculated based on the maximum value of force supported (F_max). Panel 1.1. was generated in using SolidWorks® 2022 (www.solidworks.com). Panel 1.2 was generated in Microsoft PowerPoint Version 2304 (https://www.microsoft.com/en-us/microsoft-365/powerpoint). Panel 1.3 was generated in MATLAB® R2022a (https://www.mathworks.com/products/matlab.html).

Field deploying devices which measure bending strength and bending stiffness often utilize similar form factors and methods to acquire these measurements. These devices typically approximate the stalk as a cantilever beam and apply a force at a specified height. To ensure the force is applied at the intended height most devices consist of a rigid, rotating, vertical bar supporting a load cell. The load cell can typically be adjusted vertically to accommodate plants of varying heights. In most cases, the vertical bar pivots about a foot plate or a fixed point on the ground. One such field-based device is the Device for Accessing Resistance to Lodging In Grains (aka DARLING) (Fig. 1 panel 1)^27,48^. During operation, the vertical bar of the DARLING rotates at the stalk's base while the force sensor applies a measured load to the specimen (Fig. 1 panel 2). An electronic sensor suite continuously records applied force and angle during the test and allows the user to record the height at which the load was applied. An example plot of data collected by the DARLING is shown in Fig. 1 panel 3. This type of plot is common to many devices, and the data is used to calculate bending strength and bending stiffness. Bending strength and bending stiffness are two of the most commonly measured quantities as they have been shown to strongly correlate with lodging resistance^19^. Bending stiffness (aka, flexural rigidity) and bending strength are defined in Eqs. (1) and (2) respectively, where *F* is applied force, Φ is the slope of the linear portion of the force/deflection curve (Fig. 1 panel 3) and* h* is load cell height (moment arm)^27^. The deflection is calculated using Eq. (3) where θ is the angular displacement measured by the DARLING.

1 $$EI=\frac{\phi {\cdot h}^{3}}{3}$$

2 $$S={F}_{max}\cdot h$$

3 $$Deflection=h\cdot \mathrm{sin}(\theta )$$

Several sources of experimental error can reduce the accuracy of data used to calculate bending strength and bending stiffness. The simplest source of error is that in the recorded load cell height (h). Perhaps less obvious is the error due to placement of the device relative to the base of the stalk. Irregularities in the surface of a field, the presence of brace roots, and user fatigue can often lead to the device pivoting either in front of the stalk or behind the stalk. In addition, when stalks undergo large deflection before breaking several other sources of error are introduced. These are explained in more detail below.

**Figure 2:**
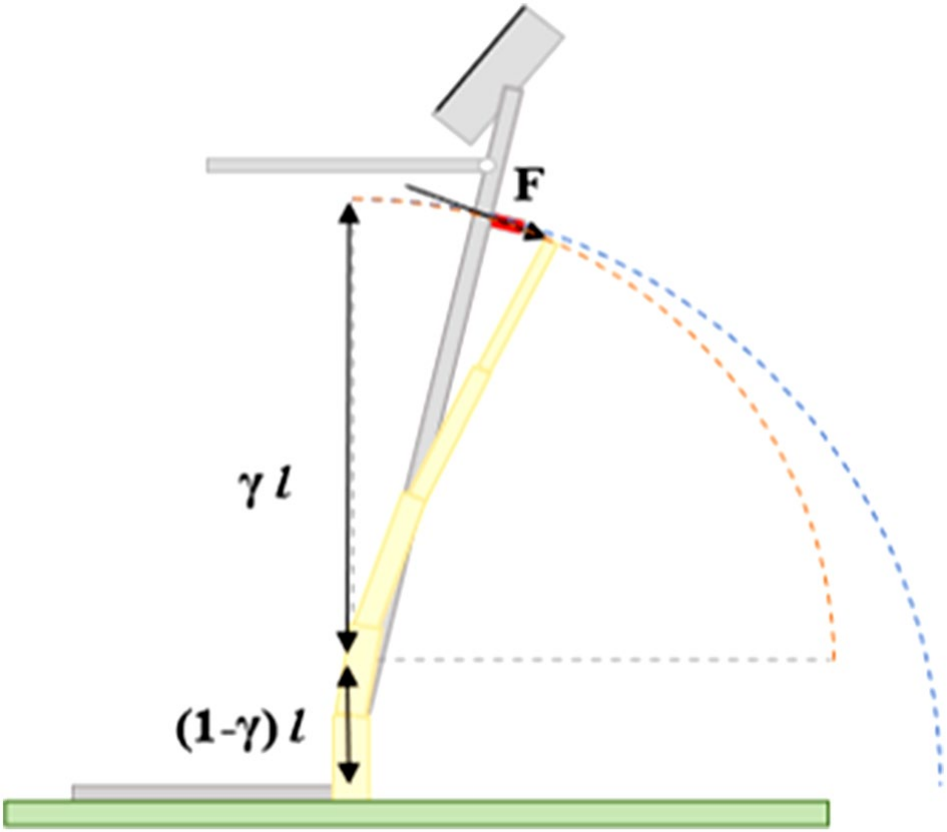
The deflected path of a plant stem is approximately circular (shown in orange), but the center of the curvature of the path is centered at some point along the stem’s length. The location of the center of curvature of the deflected path is often denoted using (γ) where γ ~ 0.15. The path of the DARLING is also circular (shown in blue) but the center of curvature of the DARLING’s path is centered at the pivot point of the darling. This difference in path center points results in path divergence as angular deflection increases. Figure was generated in Microsoft PowerPoint Version 2304 (https://www.microsoft.com/en-us/microsoft-365/powerpoint).

**Figure 3:**
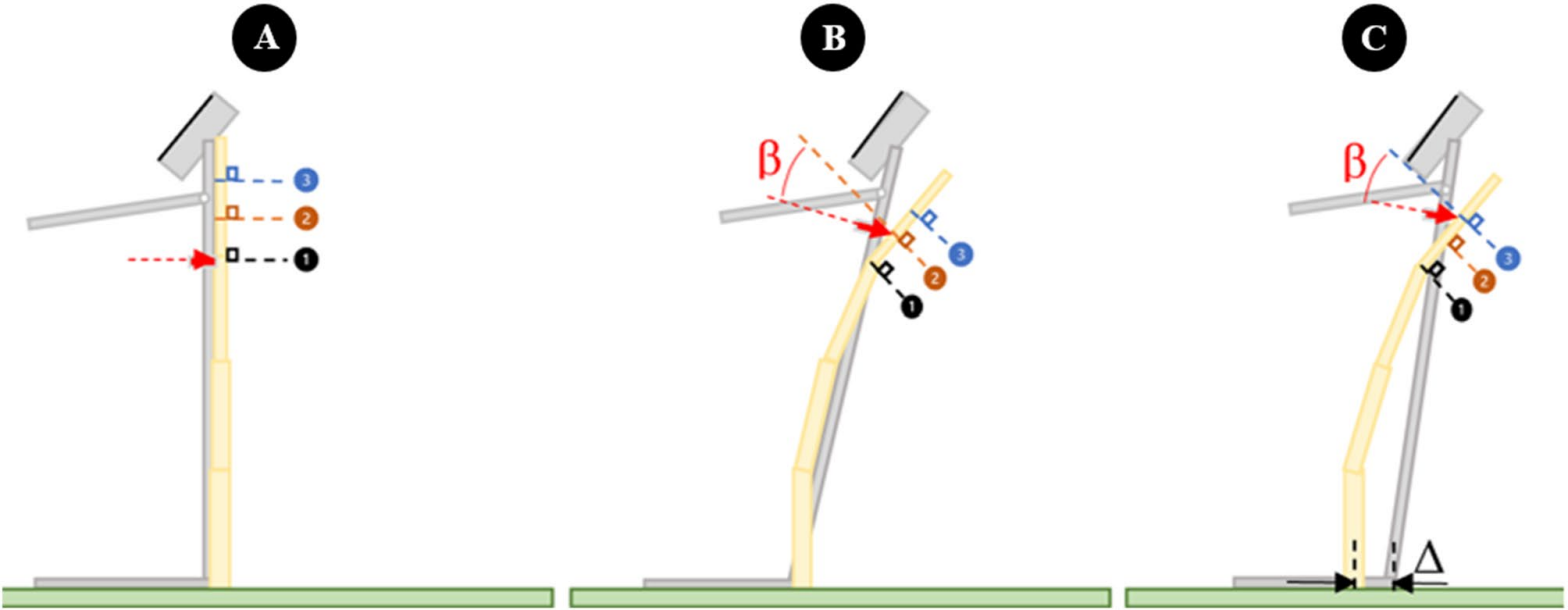
Illustration of load cell sliding along the length of a stalk during testing. Panel (**A**) shows the undeflected stalk. Panel (**B**) shows a deflected stalk in which the DARLING was properly aligned at the base of the stalk. Note that even when properly aligned with the base of the stalk the load cell will still slide along the length of the stalk and will not remain perpendicular to the deflected stalk. Panel (**C**) illustrates a deflected stalk in which the DARLING was not properly aligned with the base of the stalk. In this case the load cell starts non-normal to the stalk. As the stalk deflects the load cell will slide up the stalk to point 2 or point 3 as illustrated in panels (**B**) and (**C**). If the device is positioned behind the stalk (+ Δ), the load cell will slide less but it will be oriented at a more obtuse angle (β). Figure was generated in Microsoft PowerPoint Version 2304 (https://www.microsoft.com/en-us/microsoft-365/powerpoint).

Plant stalks behave comparably to classical cantilever beams and engineering beam theory is frequently used to calculate mechanical properties of stalks during in-field phenotyping tests. For example, Eq. (1) comes directly from Euler Bernoulli beam theory. When a cantilever beam subjected to a follower load undergoes large deflections (> 10°) the deflected path of the end of the beam is approximately circular. However, the center of curvature of this path is centered at some point along the length of the beam and not at its base^49^. This phenomena is well known and the center of the curvature (often referred to as the characteristic pivot) generally resides at 15% of the length of the beam, measured from the fixed end^49,50^ (Fig. 2). However, field-based phenotyping devices often pivot at ground level (i.e., base of the stalk). Discrepancies between the location of the device's pivot point and the plant’s characteristic pivot cause the load cell to slide along the length of the stalk as the plant is deflected (see Fig. 3). When this occurs, the load cell axis will not remain perpendicular to the stalk. This is problematic as the type of load cells used in these devices are designed to measure normal loads only. When the load cell is no longer perpendicular to the stalk non normal loads are introduced, creating error in force measurements. Discrepancies between the location of the device's pivot point and the plant’s characteristic pivot also introduce error into angular deflection measurements. The effect of these errors on calculations of bending stiffness and bending strength have yet to be quantified.

To quantify the amount of error introduced by (1) inaccurate placement of the device pivot relative to the base of the stalk, (2) the characteristic pivot phenomena and (3) inaccurate load cell heights, an artificial maize stalk was created and submitted to a barrage of experimental tests using a DARLING device. The systematic error and random error present in each test were calculated.

## Methods

**Figure 4:**
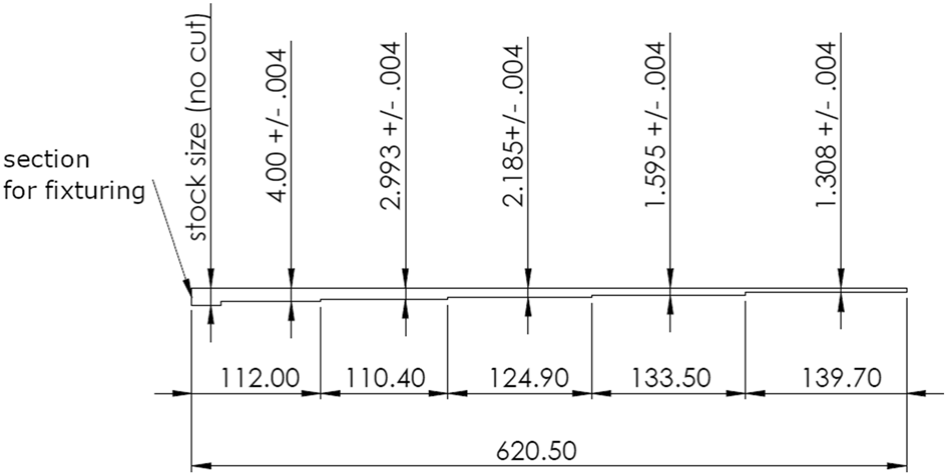
An artificial inbred maize stalk was constructed from a protruded carbon fiber rod. The dimensions of the beam shown above were determined based on the average stalk geometric ratios of 200 stalks from an inbred data set. Units are in mm and the drawing is not shown to scale to increase definition and clarity.

When subjected to external loading, plant stems often exhibit viscoelastic behaviors that can change with moisture content and time of day^51^. Therefore, in this study we created an artificial stalk specimen that could be repeatedly tested and reliably provide the same mechanical response over time. To create this artificial stalk, we analyzed a data set of 200 inbred maize stalks. The internode lengths, and the moment of inertia of the stalks were used to inform the construction of a protruded carbon fiber rod. In particular, the relative reduction in moment of inertia along the length of the rod was proportional to the reduction in moment of inertia along the length of an average inbred maize stalk. This ensured the rod would deflect in a similar manner to an average inbred maize stalk from our dataset. The exact dimensions of the rod are shown in Fig. 4.

**Figure 5:**
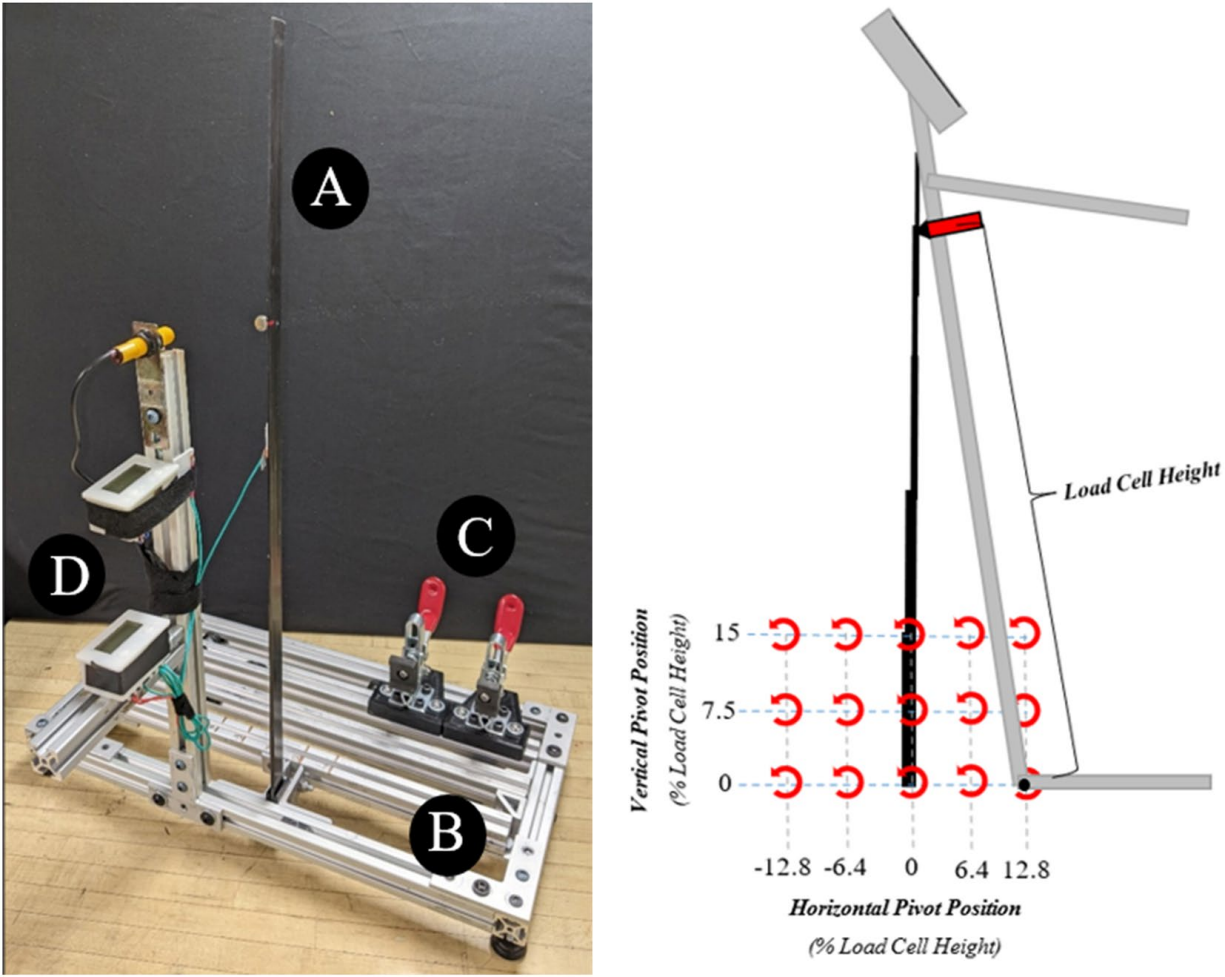
(Left) Carbon fiber stalk (**A**) fixtured into test frame. The frame consists of an aluminum skeleton (**B**), toggle clamps used to secure the foot of the DARLING in place and (**C**) a deflection sensor system (**D**). (Right) Position of the DARLING pivot relative to the carbon fiber rod for each of 15 locations. Ten tests were performed at each testing location, resulting in 150 total tests. Note that visual spacing between axis tick lines is exaggerated to increase definition. Right hand panel generated in Microsoft PowerPoint Version 2304 (https://www.microsoft.com/en-us/microsoft-365/powerpoint).

An aluminum test fixture, shown in Fig. 5: Left panel, enabled the DARLING to be positioned relative to the base of the protruded rod in both the vertical and horizontal directions. Tests were performed at five horizontal positions (± 6.4%, ± 12.8%, 0% of load cell height) and three vertical positions (0%, 7.5%, 15% of load cell height) as shown in the right-hand panel of Fig. 5. At each position, 10 tests were performed. This resulted in a total of 150 tests (five horizontal positions x three vertical positions × 10 tests at each position = 150 tests). During each test a proximity sensor alerted the user when the stalk was deflected to 10 degrees. At this point, the user returned the DARLING to the upright position and then deflected the stalk again to 25° at which point another sensor alerted the user. The DARLING was then returned to the upright position and the test was stopped. Note that the test fixture sensors were used to detect stalk deflection (and not DARLING deflection). In other words, even though the horizontal and vertical positions of the DARLING were changed throughout the study, the stalk was deflected to the same two points in every test. The portion of the test in which the stalk was deflected to 10° was used to determine the bending stiffness of the rod. This is standard practice as the load deflection curve was linear below 10 degrees of deflection. The load at 25° of stalk deflection was used as a surrogate bending strength measurement. A custom MATLAB program^27^ was used to determine flexural stiffness and bending strength of the protruded rod in each test as described in Ref.^27^ using Eqs. (1, 2, 3).

After computing bending strength and flexural stiffness measurements the systematic error in each measurement was calculated. The systematic error was defined as:

4 $$\%\,error\,S= \frac{{{ S}_{measured}-S}_{actual}}{{S}_{actual}}\cdot 100$$

5 $$\%\,error\,EI= \frac{{{ EI}_{measured}-EI}_{actual}}{{EI}_{actual}}\cdot 100$$

where S_actual_ and EI_actual_ were the average measured values of S and EI from ten tests performed at zero horizontal offset and 15% vertical offset. At this position the DARLING pivot is closely aligned with the characteristic pivot of the protruded carbon fiber rod and the load cell does not slide along the length of the stalk during the test. The relative standard deviation at each testing position was calculated to determine the presence of random error, where:

6 $$RSD=\frac{\sigma }{\overline{m} }\cdot 100$$

With σ representing standard deviation and $\overline{m }$ representing the mean of the ten tests performed at each test location.

To determine the effects of erroneous load cell height on calculations of bending stiffness and bending strength these quantities were recalculated for the data set described above using incorrect load cell heights. More specifically, all 150 tests were performed at a load cell height of 47 cm. However, to determine the effects of erroneous load cell heights the load displacement-data from the 150 tests were analyzed to compute bending strength and flexural stiffness (see Eqs. 1, 2, 3) while assuming erroneous load cell heights of 32 cm, 46 cm, 48 cm and 62 cm (e.g., the value of h in Eq. (1) was set to 32 cm, 46 cm, 48 cm, and 62 cm instead of 47 cm). The % error in EI and the % error in S due to erroneous load cell heights were then calculated.

In summary, systematic and random error was calculated at 15 different test positions as shown in Fig. 5. These test positions were selected based on the authors’ prior experience utilizing phenotyping devices. We estimate that it is common for users to horizontally misalign the pivot of a phenotyping device by ± 3 cm which corresponds to a 6.4% offset if the load cell height is 47 cm. A 47 cm load cell height is typical when testing inbred maize stalks. We estimate that horizontally misaligning the device pivot by ± 6 cm (i.e., 12.8% of a 47 cm load cell height) is quite noticeable to most users. However, this is also somewhat common due to user fatigue and variations present in the field environment (e.g., uneven ground and other plants obstructing the phenotyping device). Horizontal placement errors greater than ± 6 cm are uncommon as beyond this range the ergonomics of the device become uncomfortable and unwieldy. We choose to select three positions for vertical offsets. A 0% vertical offset is the norm for most phenotyping devices. A 15% vertical offset was chosen as it is the approximate location of the characteristic pivot of a prismatic cantilever beam. A 7.5% vertical offset was chosen simply because it evenly separated the 0% and 15% offsets. The exact position of the characteristic pivot has not been precisely determined for stepped cantilevered beams though it is assumed to be slightly greater than 15%. Further research into the exact position of the characteristic pivot position of stepped cantilevered beams is required to calculate systematic error more accurately. The magnitudes of systematic error presented in this study should therefore be viewed as the minimum possible value. The values of load cell height utilized were also chosen based on the author’s experience. The DARLING device has a ruler engraved on it that is separated into 15 cm increments. We have observed that users typically align the load cell precisely with these markings. However, an exceptionally careless user may place the load cell 1 cm above or below a mark. A far more common type of error is for a diligent but tired user to move the load cell up or down by a 15 cm increment and forget to input the new load cell height into the DARLING. These observations led us to choose four erroneous load cell height measurements: 46 cm and 48 cm (error of ± 1 cm), as well as 37 cm and 62 cm (error of ± 15 cm).

A limited number of field tests were also conducted as part of this this study to demonstrate that the carbon fiber rod was a reasonable proxy for a maize stalk. In particular, ten corn stalks were subjected to repeated, nondestructive flexural test using a DARLING device that was placed in several different positions relative to the base of the stalk. The load cell height during these tests was 75 cm (as opposed to 45 cm). Tests were performed at six different horizontal positions (0%, 3.4% 6.8%, 10.2%, 13.6% 16.9% of load cell height). There was a 0% vertical offset for each test. Each stalk was tested at each position and the raw force displacement data was analyzed to calculate flexural stiffness as described above. The % error in flexural stiffness between the 0% horizontal offset test position and each of the other test positions was then calculated and compared to the results from the lab-based experiments performed using the carbon fiber rod.

## Results

**Figure 6:**
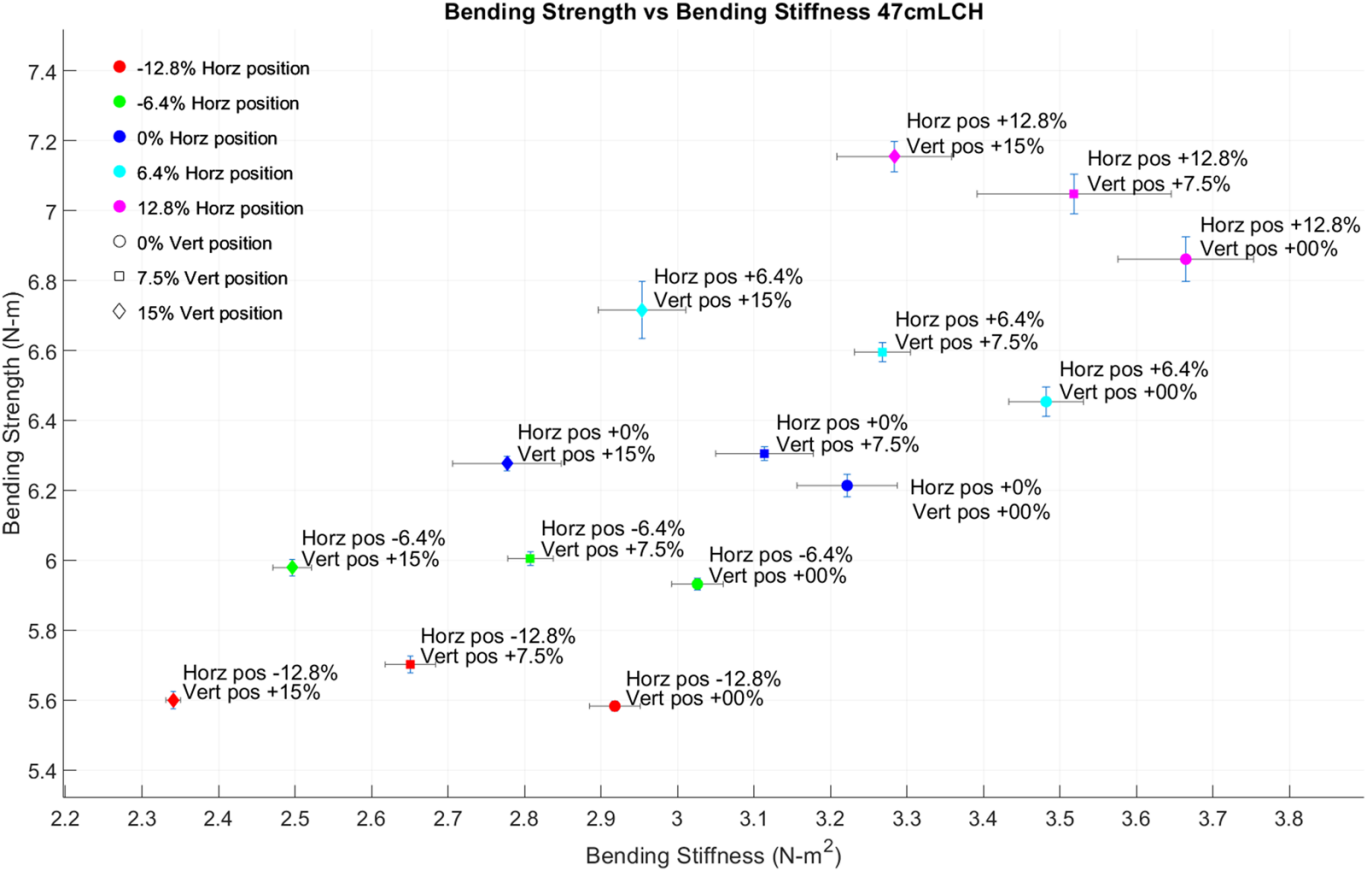
The average bending stiffness and bending strength obtained at each testing position. Error bars are 1 standard deviation in length in both the EI and S axes. Marker color indicates horizontal position, while marker shape indicates vertical position.

Of the 150 lab-based tests performed on the carbon fiber rod, two were excluded due to excessive noise. Results from the remaining 148 tests demonstrated that horizontal placement of the DARLING affected both bending strength and bending stiffness measurements. Vertical placement of the DARLING affected bending stiffness measurements but had a minimal effect on bending strength measurements. The systematic error in stiffness measurements was highest at + 12.8% horizontal offset and 0% vertical offset. Relative standard deviation was used to determine the amount of random error present at each testing position. The random error in bending strength measurements was typically below 1%. For both bending stiffness and bending strength, pivoting at positions less than 15% of the load cell height tended to increase random error. Figure 6 displays the mean bending strength and bending stiffness obtained at each testing position. Table 1 displays the percentage error (systematic error) and relative standard deviation (random error) of bending strength and bending stiffness measurements at each testing position.

**Table 1:** Percentage (systematic) error and relative standard deviation (random error) in bending strength (S) and bending stiffness (EI) measurements at 15 testing positions for a 47 cm load cell height.

	Horizontal position (% load cell height)	Vertical position (% load cell height)	Error in bending strength (%)	Error in flexural stiffness (%)	Relative standard deviation in bending strength (%)	Relative standard deviation in flexural stiffness (%)
Testing position	− 12.8	0	– 11.1	5.0	0.3	1.1
	− 6.4	0	– 5.6	9.0	0.3	1.1
	0	0	– 1.1	15.8	0.5	2.0
	6.4	0	2.7	25.2	0.7	1.4
	12.8	0	9.2	31.7	0.9	2.4
	− 12.8	7.5	– 9.2	− 4.7	0.4	1.2
	− 6.4	7.5	− 4.5	1.1	0.3	1.1
	0	7.5	0.5	11.9	0.3	2.1
	6.4	7.5	5.1	17.6	0.4	1.1
	12.8	7.5	12.3	26.6	0.8	3.7
	− 12.8	15	− 10.8	− 15.8	0.4	0.4
	− 6.4	15	− 4.8	− 10.1	0.4	1.0
	0	15	0.0	0.0	0.3	2.6
	6.4	15	7.0	6.1	1.2	1.9
	12.8	15	13.9	18.0	0.6	2.3

The first two columns define the testing position of the device relative to the base of stalk (see Fig. 5 for visual description of all testing positions). The other four columns express the systematic and random error for each testing position.

**Figure 7:**
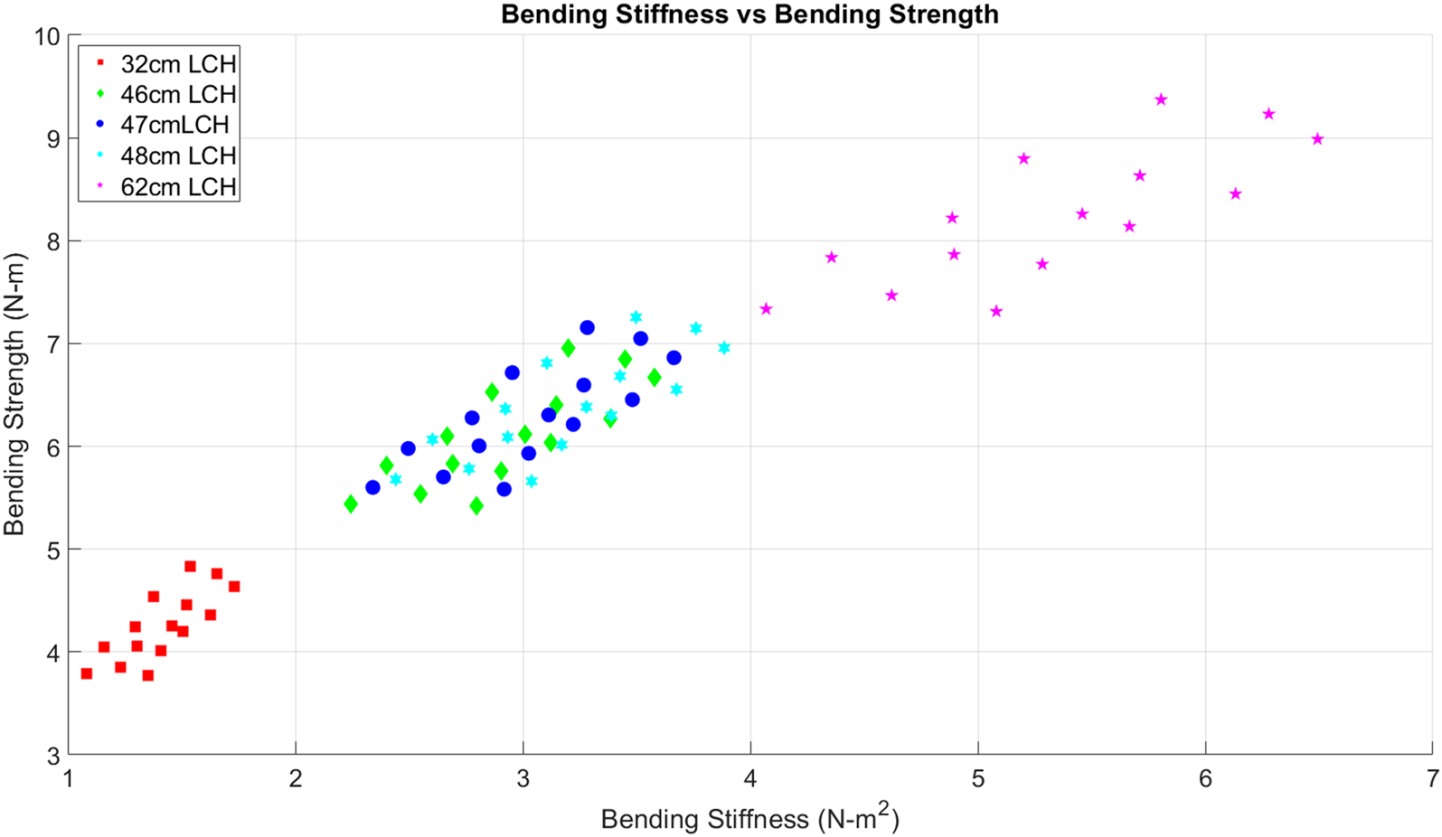
Spread of bending stiffness and bending strength measurements obtained while using erroneous load cell heights. Each colored data set consists of average bending stiffness and bending strength values at each of the 15 testing positions mentioned previously.

Error due to incorrect load cell height was investigated by recalculating bending stiffness and bending strength for all 148 tests using incorrect load cell heights of 32 cm, 46 cm, 48 cm and 62 cm. The actual load cell height during the experiments was 47 cm. The systematic error in bending strength measurements was directly proportional to error in load cell height when the horizontal offset was 0%. This was expected considering the linear dependency of bending strength on load cell height (see Eq. (2)). However, at other horizontal and vertical offsets the resultant error was less predictable. In general, erroneous load cell height values had a more drastic impact on bending stiffness measurements than on bending strength measurements (see Table 2 and Fig. 7). This was expected as bending strength is proportional to load cell height whereas bending stiffness is proportional to load cell height raised to the third power (see Eqs. (1) and (2)). For example, when an incorrect load cell height of 32 cm was utilized bending strength errors ranged from − 23 to − 40% while flexural stiffness errors ranged from − 40 to − 60% (Table 2). When an incorrect load cell height of 62 cm was utilized bending strength errors ranged from 16 to 49% while flexural stiffness errors ranged from 46 to 133% (Table 2).

**Table 2:** Percentage error for bending strength (S) and bending stiffness (EI) at 15 testing positions for erroneous load cell heights (LCH) of 32 cm, 46 cm, 48 cm, and 62 cm.

	Horizontal position (% of 47 cm load cell height)	Vertical position (% of 47 cm load cell height)	S error (%)					EI error (%)				
			32 cm LCH	46 cm LCH	47 cm LCH	48 cm LCH	62 cm LCH	32 cm LCH	46 cm LCH	47 cm LCH	48 cm LCH	62 cm LCH
Testing position	− 12.8	0	− 39.9	− 13.6	− 11.1	− 9.8	16.5	− 51.2	0.8	5.1	9.4	83.0
	− 6.4	0	− 36.1	− 8.2	− 5.5	− 4.1	23.8	− 49.2	4.7	9.0	14.2	90.3
	0	0	− 33.1	− 3.8	− 1.0	0.4	29.7	− 45.8	12.4	16.0	22.0	104.1
	6.4	0	− 30.5	− 0.1	2.8	4.4	34.7	− 41.4	21.9	25.4	32.3	120.8
	12.8	0	− 26.1	6.2	9.3	10.9	43.2	− 37.7	28.9	32.0	39.9	133.8
	− 12.8	7.5	− 38.6	− 11.7	− 9.2	− 7.9	19.0	− 55.6	− 8.2	− 4.5	− 0.5	66.4
	− 6.4	7.5	− 35.3	− 7.0	− 4.3	− 3.0	25.3	− 53.1	− 3.1	1.1	5.6	76.3
	0	7.5	− 32.2	− 2.5	0.5	1.7	31.6	− 47.6	8.4	12.1	18.1	96.6
	6.4	7.5	− 29.0	2.1	5.1	6.5	37.5	− 45.2	13.3	17.7	23.4	105.7
	12.8	7.5	− 24.1	9.1	12.3	13.9	47.1	− 40.4	24.2	26.7	35.4	126.1
	−12.8	15	− 39.7	− 13.3	− 10.8	− 9.5	16.9	− 60.9	− 19.2	− 15.7	− 12.1	46.5
	− 6.4	15	− 35.6	− 7.4	− 4.7	− 3.3	24.8	− 58.3	− 13.6	− 10.1	− 6.3	56.9
	0	15	− 32.4	− 2.8	0.0	1.4	31.0	− 53.3	− 3.9	0.0	5.3	76.0
	6.4	15	− 27.7	4.0	7.0	8.5	40.2	− 50.4	3.1	6.4	11.8	87.3
	12.8	15	− 22.9	10.8	14.0	15.6	49.3	− 44.6	15.2	18.2	26.0	109.1

**Figure 8:**
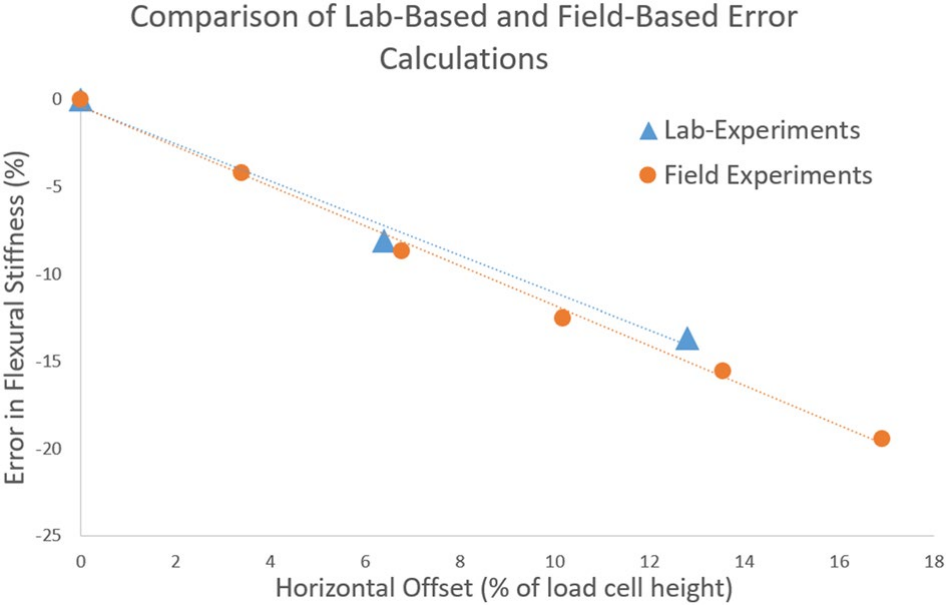
Comparison of lab-based and field-based error calculations for flexural stiffness showed strong agreement indicating that the machined carbon fiber rod used in the study adequately mimicked the flexural response of a maize stalk.

Field-based testing of 10 mature maize stalks confirmed the results of the lab-based test performed on the carbon fiber rod. In particular, the error in flexural stiffness expressed as a function of horizontal offset for the field-based tests were very close to the error estimates obtained from the lab-based study. Figure 8 displays the % error in flexural stiffness vs horizontal offset for both the lab-based (carbon fiber rod) and field-based (mature maize stalks) data. Note that in this figure the % error in flexural stiffness is calculated relative to 0% horizontal offset and 0% vertical offset whereas the % error displayed in Tables 1 and 2 were calculated relative to 0% horizontal offset and 15% vertical offset. This is because none of the field-based test were performed with a 15% vertical offset due to difficulties associated with trying to raise the DARLING off the ground in the field.

## Discussion

The lodging resistance of individual crop varieties has traditionally been assessed through natural observations of lodging. Experimental units (e.g., plots) are often observed just prior to harvest and subjectively scored. Aerial imaging techniques have been applied more recently to quantify the % area of lodged crops to provide a more quantitative metric. The largest challenge with this approach is that some experimental units will be affected by more extreme weather events than others and therefore experience more lodging. In addition, during good growing conditions none of the varieties may experience lodging and therefore no determination of the relative lodging resistance of the different varieties can be made. Thus, relying on natural lodging incidence to differentiate the lodging susceptibility of crop varieties requires extensive field studies that span multiple years and locations and are often cost prohibitive. Biomechanical phenotyping tools like the DARLING are attractive because they can phenotype an experimental unit for bending strength or bending stiffness in the absence of natural lodging. Thus, even in optimal growing conditions when no natural lodging occurs such phenotyping devices can differentiate varieties for lodging resistance. For example, it was recently shown that DARLING measurements obtained for 47 hybrids in just three environments were able to accurately predict the historical rates of natural lodging incidence of those 47 hybrids obtained in 98 distinct environments spanning four years and 41 unique geographical locations in North America^6^.

Bending strength and bending stiffness are primary determinants of stalk lodging resistance^6,52,53^. Unfortunately, significant amounts of human labor are required to attain measurements of bending strength and bending stiffness. The cost of attaining these measurements is a challenge and phenotyping for these traits is a bottleneck limiting genetic improvement of stalk lodging resistance^54,55^. Systematic and random error present in field-based measurements of bending stiffness and bending strength exacerbates this issue. One way to partially mitigate systematic and random error is to increase the number of sampled plants in a study and to calculate average or median values for each variety included in the study. Unfortunately, increasing sample size requires additional human labor inputs. Therefore, in this study we sought to identify principal sources of error present in field-based measurements of stalk bending strength and bending stiffness so that they may eventually be rectified. This is the first error analysis of any field based biomechanical phenotyping methodology of which the authors are aware.

Results from this study as well as prior experience using the DARLING device over several years suggest that improvements can be made to operating procedures and phenotyping devices to mitigate systematic errors in bending strength and bending stiffness measurements. When spending long hours in the field collecting phenotyping data it is common to make mistakes when recording load cell height. In particular, we have found that users sometimes forget to record a new load cell height in the DARLING software after physically changing the height of the load cell. This can produce very large systematic error (> 100%) in bending strength and bending stiffness measurements. This type of error can sometimes be detected during post processing. Bending strength and bending stiffness are highly correlated and typically have an R^2^ value near 0.7. Bending strength varies linearly with load cell height but bending stiffness varies with load cell height raised to the third power. Therefore, test conducted at an incorrect load cell height will often show up as outliers in a scatter plot of bending strength vs bending stiffness. Unfortunately, while this can help to identify incorrect load cell heights there is generally no way to reliably correct the error with confidence. Additional user training and standardizing operating protocols can alleviate but not fully eliminate this source of error. Including a load cell height sensor on the device that automatically records load cell height, or that notifies the user when the load cell has been changed is a promising approach to eliminate this source of systematic error in the future.

The amount of systematic error introduced by incorrect horizontal placement of the phenotyping device pivot can be partially mitigated by using the highest reasonable load cell height. The amount of systematic error produced by horizontal offsets is a function of the horizontal offset expressed as a percentage of load cell height. Thus a 3 cm horizontal offset will produce less systematic error when the load cell height is 75 cm than when the load cell is 45 cm. We believe this source of systematic error can also be mitigated by properly training and explaining the effects of horizontal placement on measurement error to device users. Over the past seven years we have used the DARLING to conduct over 200,000 bending tests and the device has been used by over 20 different research groups. We found that most users of biomechanical phenotyping devices do not have engineering backgrounds and the importance of device placement is not intuitive. In our experience it has been crucial to spend a full day conducting in-person, field-based training of new users of the DARLING device to ensure quality data is collected. Virtual training of new users has always resulted in poor data quality and has sometimes resulted in entire studies being discarded. Horizontal placement error could potentially be mitigated by adding an extra feature to phenotyping devices to help the user ensure the phenotyping device pivot is correctly aligned with the stalk. However, the constantly varying conditions found within agricultural plots (e.g., uneven ground, brace roots, adjacent plants etc.) make this a nontrivial design challenge. It’s the authors opinion that the most promising approach to rectifying this error is proper training.

More complex modifications to testing equipment could minimize systematic error due to vertical discrepancies between the device pivot and the characteristic pivot of the plant being tested. Ideally the device would pivot at the characteristic pivot height of each plant. However, the characteristic pivot height is a function of the height at which the load is applying a force to the stalk. Thus, the pivot height would have to change every time the load cell height was changed. Requiring users to manually change the pivot height would significantly reduce throughput. Alternatively, a mechanical linkage could be designed that would change the pivot height whenever the load cell height was changed. This linkage would increase device cost, and weight which would in turn increase user fatigue. Alternatively, one could attempt to account for and correct systematic errors due to vertically misaligned pivot points during data post processing. This is a promising approach and is supported by the fact that we observed strong agreement between horizontal placement error of in-field bending stiffness tests and lab-based bending stiffness test. In other words, the results from the lab-based portion of this study predicted the actual error during in-field bending stiffness measurements. However, to more accurately correct error due to vertical placement additional research into the large deflection response of stepped cantilever beams is needed. Finding other ways to reduce systematic error are warranted as they will enable future researchers to utilize reduced sample sizes (and human labor inputs) in phenotyping trials.

Results indicated that random error was also a function of testing position. Random error was quantified by calculating the relative standard deviation (aka coefficient of variation) at each test location. Both random error and systematic error increase the sample size required to attain reliable average bending strength and bending stiffness values of plant varieties of interest. While the magnitude of random error was less than that of systematic error it still warrants attention. Results demonstrated that testing at positive horizontal pivot positions (Fig. 5) resulted in the largest values of relative standard deviation for both bending stiffness and bending strength. Testing at negative horizontal positions minimized the random error but should be avoided as it introduces significant systematic error. Like systematic error, the random error was minimized when the device pivot and characteristic pivot of the stalk were vertically aligned. However, as described previously the technical challenges associated with vertical aligning the pivots for every tested sample may outweigh its benefit.

Several other sources of error are present in field based biomechanical measurements of plants stems that were not investigated in this study. For example, if plants are rapidly deflected inertial effects can introduce additional forces that are detected by the load cell. The measured force can also be significantly altered if the stalk being tested contacts adjacent plants or the ground during the test. In addition, if the top section of the stalk is not removed prior to testing it can oscillate during the test which introduces error in the measured force. To prevent the tested stalk from contacting adjacent plants the leaves and the top portion of the stalk are often removed immediately prior to testing^56^. When doing so care should be taken to either leave the leaf sheath completely intact or to remove it completely. Several studies have shown that the leaf sheath contributes significantly to bending strength and bending stiffness^57,58^. The DARLING and other similar devices often assume the stalk is rigidly anchored in the soil. However, if the soil is loose or wet the stalk and root structure may rotate in the soil. This does not alter bending strength measurements, but it can drastically alter bending stiffness measurements. Lastly, any electronic or analog sensor has inherent limits, resolution, and accuracy. Low-cost sensors are appealing but they are often unreliable. For example, low-cost load cells are widely available, but their readings can be significantly affected by temperature, relative humidity, and electronic noise. Simultaneously accounting for and mitigating all these sources of error can be especially challenging. Ideally researchers with agricultural, genetic, or biological backgrounds should collaborate with individuals who possess expertise in metrology, or engineering when conducting biomechanical phenotyping studies. Doing so will improve the accuracy and reliability of measured quantities (e.g.,^59,60^). Metrology and engineering expertise can also be leveraged to establish regular and standardized calibration routines for biomechanical phenotyping devices.

While it is impossible to precisely quantity, we estimate that errors on the order of 15–25% in bending stiffness and 1–10% in bending strength measurements are not uncommon in biomechanical phenotyping studies utilizing a DARLING device. These estimates are admittedly subjective and are based on our experience training many different users ranging from undergraduate and graduate students to field technicians and professional scientists, in a variety of locations and conditions over the course of seven years. A minimum error of approximately 1% in bending strength and 15% in bending stiffness is expected in all studies. These minimum values can be obtained from Table 1 and correspond to a test with zero horizontal offset, zero vertical offset and no error in the load cell height. This minimum error is cue to the DARLING device’s pivot being at ground level and not at the characteristic pivot of the plant being tested. The upper bounds of our error estimates for bending strength and bending stiffness were made by observing the results from the top 5 rows of Table 2 (zero percent vertical offset) and considering the following. Some horizontal test positions produce positive error while others produce negative error. As multiple tests (5–10) are typically conducted in each plot and plot averages are then used for analysis these errors will partially cancel each other out. However, we have observed that users tend to positive horizontal test positions more than negative horizontal positions as negative positions are ergonomically disfavored. We believe the majority of tests in any study are conducted with less than 1 cm of error in load cell height. However, during an eight-hour testing session a user may forget to input a new load cell height into the DARLING software two or three times. Users often realize this after a few tests and make a note so the error can be corrected later. However, sometimes they are not sure how many tests have been conducted since the load cell height was changed. This is a challenging situation to rectify but is sometimes possible when post processing the data. We suspect that less than 1% of tests may be conducted at an incorrect load cell height that do not get excluded during post processing. In addition, many stalks are tested at heights greater than 47 cm thus the amount of error produced is less than the values presented in Table 2. In other words, we have observed that while error is dependent upon the offset expressed as a percentage of load cell height, the accuracy of users in placing the device and inputting the load cell height is not dependent upon load cell height. They typically place the device within 6 cm of the base of the stalk regardless of load cell height. Considering these factors and examining the many studies we have been involved with that used a DARLING device we give a broad estimate of the expected upper bounds of error in bending strength and bending stiffness as 10% and 25% respectively. Note that as with any experimental technique much higher errors can be produced with improper training or careless data collection.

Errors on the order of 15–25% in bending stiffness and 1–10% in bending strength may appear large and there is certainly room for improvement. However, we expect even larger errors are present in most of the subjective phenotyping methods used to characterize lodging, including natural lodging incidence. In addition, these are estimates of the absolute error (deviation from the actual bending stiffness and bending strength). All DARLING measurements have a minimum absolute error of 15% error in bending stiffness due to the characteristic pivot phenomenon. However, this error has little effect on the determination of lodging resistance vs lodging prone varieties as it is present in every test and therefore does not affect relative rankings between varieties.

The primary factor which governs the flexural deformation of any solid structure (be it natural or manmade) is the section modulus. The carbon fiber rod used in this study was machined so that it possessed the section modulus of an average inbred maize stalk. Thus, while the material arrangements of maize plants and the machined carbon fiber rod used in this study are quite different, the in-field and lab-based error analysis results showed very strong agreement. It is possible that at very large deformations and near the point of failure other factors may alter the flexural response of plants. For example, it has been suggested that plant stems may undergo brazier buckling (deformation of the cross-sectional geometry) just prior to failure. Due to differences in material arrangement Brazier buckling would not occur in the carbon fiber rod. The authors believe that these other factors would not alter the fundamental finding of this study. The findings of the study are grounded in sound engineering principles that govern the flexural response of structures. We therefore expect that other crops with similar anatomical and structural arrangements to maize (e.g., sorghum, sugar cane) would exhibit similar errors to those found in this study. In other words, we believe these results are generally applicable to the DARLING device. However, we recommend caution in extrapolating these results to other measurement devices. The authors have overseen the testing of over 200,000 plants with the DARLING device and are very familiar with its operation and intricacies. Other devices may exhibit sources of errors which were not characterized in this study and which the authors could not anticipate without using such devices extensively.

## Conclusion

To the best of the authors’ knowledge, this is the first formal study which attempted to quantify systematic and random error present in field-based phenotyping methodologies used to quantify bending strength and bending stiffness of plant stems. We conclude that significant amounts of error can easily be introduced when conducting field based biomechanical measurements of plant stems. We estimate that errors on the order of 15–25% in bending stiffness and 1–10% in bending strength measurements are not uncommon when using a DARLING device. This error can be mitigated by following best practices and strict operating protocols to ensure that the pivot of the phenotyping device is aligned with the characteristic pivot the stalk being measured. Several design improvements can also be made to current phenotyping devices that would reduce both systematic and random error in biomechanical measurements. Future research in this area is warranted and could lead to further genetic improvements in stalk lodging resistance.

## Data Availability

All datasets used and/or analyzed during the current study are available from the corresponding author on reasonable request.
